# A Case of Rapidly Progressive Dementia

**DOI:** 10.7759/cureus.22507

**Published:** 2022-02-22

**Authors:** Arun Thekkekarott Kuruvila, Nishant Ranawat, Nikita Hegde, Alok Arora

**Affiliations:** 1 Hospital Medicine, Asante Rogue Regional Medical Center, Medford, USA; 2 Neurology, Asante Rogue Regional Medical Center, Medford, USA; 3 Anaesthesia, A. J. Institute of Medical Sciences, Mangalore, IND; 4 Internal Medicine, Aurora Medical Center-Bay Area, Marinette, USA

**Keywords:** rapid eye movement sleep, rapid dementia, creutzfeldt jakob disease, rapidly progressive dementia, hemichorea, dystonia, bovine spongiform encephalopathy, variant creutzfeldt-jakob disease, prion diseases

## Abstract

Creutzfeldt-Jakob disease (CJD) is a very rare neurodegenerative disorder that usually presents as rapidly progressive dementia with an extremely poor prognosis. The diagnosis of CJD can be extremely challenging due to its rarity, manifestation with non-specific neurological symptoms, associated broad differentials, and a need for extensive workup. Awareness of disease-specific biomarkers, radiological signs, and diagnostic criteria are crucial for timely diagnosis. Here, we report a case of CJD, which presented as an atypical movement disorder that progressed to dementia and failure to thrive within a few weeks of presentation.

## Introduction

Creutzfeldt-Jakob disease (CJD), also known as subacute spongiform encephalopathy, is a rare, life-threatening neurodegenerative disorder. CJD is caused by the pathological misfolding and aggregation of the prion protein (PrP), a cellular glycoprotein [1]. The majority of the CJD are sporadic (approximately 85% patients) with no recognizable transmission pattern. Still, they can be due to inherited mutations of the PrP gene in a smaller proportion of patients (5% to 15%) [2]. CJD can present clinically as rapidly progressive dementia. Once transmitted to individuals, the pathologic PrP misfolds the normal PrP resulting in progressive disease. Initial manifestations include impaired memory, behavioral disturbances, visual disturbances, and poor coordination. As the disease advances, patients develop symptoms of dementia, involuntary movements, loss of vision, weakness, and coma.

## Case presentation

A 70-year-old female presented to the emergency room with complaints of involuntary movements of the left upper extremity of 10 days duration. Medical history is notable for Stanford type-A aortic dissection status post aortic repair and bioprosthetic aortic valve replacement 18 months ago. According to the patient, involuntary movements of the left upper extremity lasted several minutes, often waking her from sleep, and resolved spontaneously. She denied any fever, chills, or headache. She was seen by a neurologist three days prior. At the neurology clinic, laboratory studies were remarkable for mild thrombocytopenia with platelets 127,000. Erythrocyte sedimentation rate (ESR) was 2, and C-reactive protein (CRP) was 0.16. Electroencephalogram (EEG) was negative for seizure activity but due to clinical suspicion for atypical seizures, she was started on Levetiracetam 1,000 mg twice daily and Divalproex sodium 500 mg twice daily. However, she developed left upper extremity weakness and presented to the hospital. Imaging studies on admission as in Table 1.

**Table 1:** Radiological workup on admission

Imaging studies on admission	
Computerized Tomography (CT) scan of the brain	No acute changes
Magnetic Resonance Imaging (MRI) scan of the brain with and without contrast	Chronic small vessel ischemic changes
CT angiogram of the chest abdomen and pelvis	Negative for aortic dissection
CT angiogram of the head and neck	No arterial stenosis or venous abnormality.
MRI scan of the brain with and without contrast with thin slices of the brainstem	No acute findings

During the hospitalization, her left upper extremity weakness progressed with the development of intermittent rigidity, myoclonic jerk-like movements mimicking hemichorea. The neurologist then recommended lumbar puncture and cerebrospinal fluid (CSF) analysis (Table 2).

**Table 2:** CSF studies on admission Venereal Disease Research Laboratory test (VDRL), Glutamic Acid Decarboxylase (GAD), John Cunningham Virus (JC virus), Polymerase Chain Reaction (PCR), cerebrospinal fluid (CSF)

CSF studies on admission	
Glucose	69mg/dL
Protein	65mg/dL
Total Nucleated cells	0
Multiple sclerosis panel	Negative
Meningitis Panel PCR	Negative
Paraneoplastic antibody Panel	Negative
VDRL	Negative
Cryptococcal Antigen, Coccidioides Ab	Negative
GAD65 Ab	Negative
JC virus PCR	Negative

Antinuclear antibody (ANA) was weakly positive (0.8 units) and the autoimmune panel was otherwise negative. Autoimmune etiology remained high on the differentials; therefore, she was given hydrocortisone 1000 mg daily and intravenous immunoglobulin for five days. We noted mild improvement in the left upper extremity weakness and involuntary movements. So, she was discharged home with outpatient neurology follow-up in eight days. At the neurology clinic, she reported word-finding difficulties and poor sleep. Her neurologist noted progressive flexor posturing, movements of the left upper extremity and described the findings as “progressive hemi-dystonia.” He started her on clonazepam, baclofen and mirtazapine. He then referred her to a movement disorder clinic at a University Hospital.

Her symptoms rapidly progressed over the next five days; she became increasingly weak, developed difficulty swallowing with abnormal movements in bilateral upper extremities and rhythmic jerking of the left arm. There was some motor agitation in the legs, and she could not ambulate.

She was then readmitted to the hospital; initial vitals and lab studies were unremarkable. Repeat lumbar puncture and additional CSF testing were done (Table 3).

**Table 3:** CSF biomarker assay on readmission Real-time quaking-induced conversion (RT-QuIC), cerebrospinal fluid (CSF)

CSF Biomarker assay on readmission	
Neuron Specific Enolase	59 ng/mL (H)
RT-QuiC	Positive
T-tau protein	3,699 pg/mL ( range 0-1,149)
14-3-3 protein	Positive

**Figure 1:**
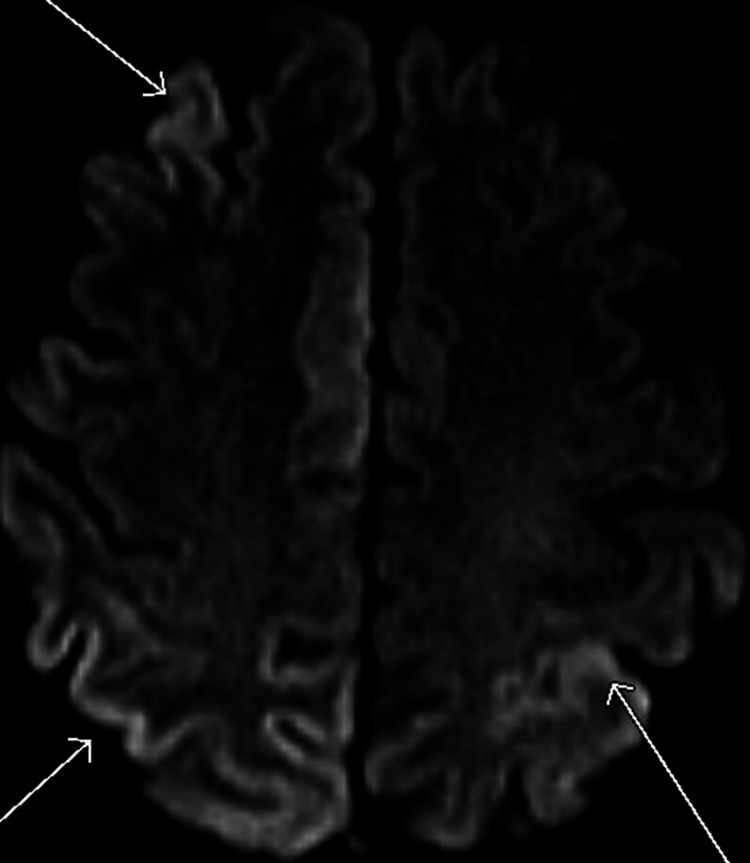
MRI brain showing cortical hyperintensity and ribboning

**Figure 2:**
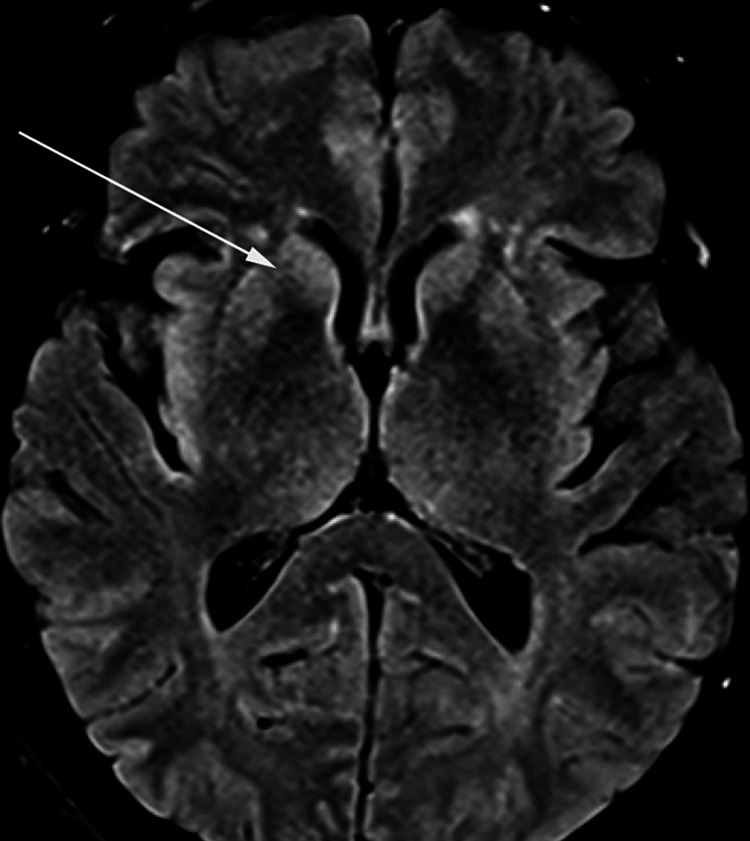
MRI brain showing hyperintensity in the caudate nucleus

A repeat MRI of the brain with and without contrast showed subtle, patchy bilateral cortical diffusion restriction, significantly progressing from prior MRIs in a pattern suggestive of CJD (Figures 1, 2). A continuous EEG showed periodic sharp wave discharges lateralized to the right hemisphere. In combination with her rapid decline, these findings were highly suggestive of sporadic CJD.

There was rapid worsening in her mentation, dementia, involuntary movements of upper extremities, and difficulty with swallowing. Given her ongoing decline and poor prognosis associated with CJD, her family decided to transition to comfort care measures. The patient passed away in the hospital on hospice, within six weeks of initial symptom onset.

## Discussion

CJD is the most common human prion disease, with an incidence of one per 1,000,000 person-years. Most of these cases are sporadic (85%-95%). The mean age of disease onset is 62 years [3-5]. Discovered initially by Creutzfeldt and Jacob in 1920, CJD was considered an atypical form of dementia until 1968 when Gibbs et al. proved experimental transmission to primates by intracerebral inoculation.

Classic clinical features of CJD are rapidly progressive dementia, ataxia, myoclonic jerks/involuntary movements [6]. The mean duration of illness is six months [7]. This rapid progression of symptoms distinguishes CJD from other common forms of dementia. Evaluation should include detailed clinical history, neuroimaging, lab studies to rule out infectious and autoimmune differential diagnoses (Table 4), electroencephalogram, and CSF analysis.

**Table 4:** Differential diagnoses

Differential diagnoses to consider when working up CJD
Alzheimer’s dementia
Dementia with Lewy bodies
Atypical meningitis/encephalitis
Autoimmune encephalitis
Paraneoplastic syndromes
Huntington’s chorea
Korsakoff Psychosis
Conversion disorder

MRI is superior to a CT scan of the brain to identify changes in CJD. A hyper-intense signal on diffusion-weighted imaging (DWI), fluid-attenuated inversion recovery (FLAIR), and T2-weighted images involving the cerebral cortex and corpus striatum, caudate head, and putamen are the most common patterns on MRI in patients with sporadic CJD [8-10]. Although a gray matter disease, CJD can affect white matter in early-intermediate stages [11].

Synchronous bi-or triphasic periodic sharp wave complexes (PSWC) on EEG can support the diagnosis in 67% to 95% of patients with CJD [12].

CSF analysis for specific markers is vital in the diagnosis of CJD (Table 5) [13-15]. The Centers for Disease Control (CDC) and Prevention have proposed diagnostic criteria for probable CJD (Table 6) and no longer recommend brain biopsy for the definitive diagnosis [16].

**Table 5:** Cerebrospinal fluid markers Enzyme-linked immunosorbent assay (ELISA), real-time quaking-induced conversion (RT-QuIC), scrapie isoform of the prion protein (PrPsc)

Cerebrospinal Fluid markers			
Test	Sensitivity %	Specificity %	To remember
RT-QuIC	95	100	Assay monitoring disease associated PrPsc transforming recombinant Prion protein (recPrP) resulting in formation of amyloid, that can be monitored in real time. The National Prion Disease Pathology Surveillance Center based at Case Western Reserve University is the only clinical laboratory in the United States that performs RT-QuIC
14-3-3 Protein	92	80	Adjunctive test; higher chance for false positives considering low prevalence of disease
Tau Protein (>1300pg/mL)	94	90	Tau-protein ELISA is easy to use in routine laboratories

**Table 6:** CDC criteria for the diagnosis of CJD Periodic sharp-wave complexes (PSWC), Centers for Disease Control (CDC), Creutzfeldt-Jakob disease (CJD)

Neuropsychiatric disorder with a positive RT-QuIC test or progressive dementia, and at least 2/4 clinical features
Myoclonus
Visual or cerebellar disturbance
Pyramidal or extrapyramidal dysfunction
Akinetic mutism
Supportive findings on one or more of the following tests
A typical EEG, e.g., PSWC during an illness of any duration
Positive 14-3-3 CSF assay with a clinical duration to death less than two years
MRI of the brain showing hyperintensity in caudate nucleus/putamen and/or in at least two cortical regions (temporal, parietal, and occipital) on DWI or FLAIR
Routine investigations should not suggest an alternative diagnosis

## Conclusions

CJD is an extremely rare disease that may manifest with a wide range of neurological symptoms as in this case. CJD is to be suspected in any case of rapidly progressive dementia or presentation with unexplained movement disorder of the limbs. A high index of suspicion, awareness of specific biomarkers, and radiologic signs are crucial in the diagnosis of this condition. Unfortunately, there are no effective treatment options for prion diseases and they are universally fatal, with a median disease duration of six months. Once a diagnosis is confirmed, physicians should provide symptomatic treatment for neuropsychiatric symptoms, communicate effectively with family regarding the biology of the disease and the expected poor outcome, and have end-of-life conversations.
